# The good, the bad, and the why: How do Arabic-speaking migrant men perceive and experience information and services related to sexual and reproductive health in Sweden?

**DOI:** 10.1016/j.jmh.2023.100153

**Published:** 2023-01-29

**Authors:** Mazen Baroudi, Isabel Goicolea, Anna-Karin Hurtig

**Affiliations:** The Department of Epidemiology and Global Health, Umeå University, Sweden

**Keywords:** Sexual and reproductive health, Cultural racism, Internalised racism, Arabic-speaking, Men

## Abstract

Although migrant men constitute a large and growing proportion of men in Sweden, literature exploring migrant men's experiences in sexual and reproductive health (SRH) services is scarce. We aimed to explore how Arabic-speaking migrant men perceive and experience information and services related to SRH in Sweden. We conducted 13 semi-structured interviews with Arabic-speaking migrant men and analysed the data using reflexive thematic analysis. We developed four themes: 1) SRH is ‘something essential in life’; 2) the good: a transition to a ‘new open society’; 3) the bad: barriers to sexual education and health services; and 4) the why: blaming oneself or the system. SRH services and sexual education/information were perceived as needs and rights, and the participants were content with the new possibilities and the ‘new open society’. However, sexual education was not provided to most migrants, and SRH services provided to men had shortcomings that deprived some migrant men from fulfilling their needs. Moreover, internalised and cultural racism created a challenge to receive adequate/acceptable SRH services. There is a need to provide comprehensive sexual education for all, strengthen SRH services provided to men, and develop an action plan to reinforce the anti-discrimination/racism policies in healthcare and society.

## Introduction

Migrants in general have worse sexual and reproductive health (SRH) compared to people born in the hosting countries (European Centre for Disease Prevention and Control, 2011; Knight et al., 2009), and they face more difficulties in accessing SRH services (Botfield et al., 2016; Mladovsky, 2009; Rechel et al., 2013). Several barriers have been documented to hinder migrants’ accessibility to SRH services in Europe including cultural barriers and stigmatisation of SRH problems, language barriers, lack of access to information about healthcare services, the mistrust in health system together with xenophobia and racism (Åkerman et al., 2016; Cha, 2013; Kalengayi et al., 2012; Svenberg et al., 2011).

Moreover, migrants usually have worse experiences receiving SRH services. Migrants visiting youth clinics in Sweden, for example, although rating the overall youth-friendliness of the clinics as high, perceived them as less friendly compared to Swedish–Scandinavian youths, particularly in the dimensions of equity, quality, and respect (Baroudi et al., 2020). In the literature, migrants’ worse experiences with healthcare have been partly explained by migrants’ social and economic status, the language and cultural differences between them and the healthcare providers (HCPs), and the lack of cultural sensitivity and the attitude of the HCPs, including cultural racism (Åkerman, 2019; Cha, 2013; Frykman, 2006; Kalengayi et al., 2012; Rafnsson and Bhopal, 2008).

Little is known about migrant men's experiences in SRH services in comparison to Swedish-born men (Baroudi et al., 2021). However, there are indications that migrant men in Sweden face more difficulties accessing and might have worse experiences receiving SRH services compared to migrant women. (Baroudi et al., 2021; Baroudi et al., 2022). Previous research showed that fewer migrant men visited SRH services (9.4% vs. 22.2% of women), and, adjusted for needs, only 23% of migrant men had their needs fulfilled by SRH services compared to 52% of migrant women (Baroudi et al., 2022). Additionally, compared to migrant women, more migrant men reported; feeling disrespected during a visit to SRH services (9.1% vs. 6.9% of women); perceiving discrimination (13.1% vs. 9.7% of women); perceiving a prejudiced attitude of the staff toward them (32.9% vs. 24.8% of women); and not receiving the expected help (30.1% vs. 20.2% of women) (Baroudi et al., 2021). Worse experiences in SRH services among migrant men are also reported in other studies from the European Union (Cha, 2013; Lebano et al., 2020). Migrant men of North African origin, for example, perceived more discrimination receiving healthcare in Italy compared to women of the same origin (Cha, 2013).

In general, there is more focus on women's SRH compared to men's SRH, creating a lower response of SRH services to men's needs (World Health Organization, 2018). In a recent review of men's experiences in SRH services in the Nordic countries, organisational factors such as SRH services being women-centred and the lower prioritisation of men's SRH were highlighted as significant obstacles to men's access to SRH services (Baroudi et al., 2021). These organisational barriers together with the societal expectations towards men to be independent and invulnerable contribute to a lower access to SRH services among men (Christianson et al., 2017; Sollesnes, 2010). These factors lead to different (generally worse) experiences of receiving SRH services among men. For example, more than half the men who visited SRH services for sexual dysfunction in Sweden reported not receiving enough help (Folkhälsomyndigheten, 2019).

Research about men's experiences in SRH services is scarce, and even less is known about migrant men's experiences in SRH services (White et al., 2011; WHO Europe, 2018; World Health Organization, 2018). In fact, a recent review of the Nordic literature about men and SRH services identified only two studies with a sole focus on migrant men related to HIV testing and men who have sex with men (Baroudi et al., 2021). To help fill the knowledge gap, this study aims to explore how Arabic-speaking migrant men perceive and experience information and services related to SRH in Sweden. Migrant men comprise a large proportion of Swedish residents; more than one million or around 20% of men in Sweden are foreign-born. Of them, more than 245 thousand or around 25% are born in the so-called Arabic world, making them the largest group of migrant men in Sweden (Statistics Sweden (SCB), 2019).

## Material and methods

### Study design and setting

This qualitative interview study took place in Sweden, with participants living in different regions around the country. Arabic-speaking men comprise the largest group of migrant men in Sweden and arrived to Sweden from various countries. Syria, Iraq, and Palestine are the most common countries of origin. They also have various ethnicities, religions, and cultures (Statistics Sweden (SCB), 2019).

In Sweden, SRH services are offered within the tax-funded public health system and include, for example, services related to maternal health, pre- and postnatal care, family planning, abortion, sexually transmitted diseases, sexual function, sexual violence, infertility, and trans-specific care. Information about sexuality and sexual health is available on public websites, such as 1177.se (national guide of health and healthcare) and umo.se (youth-oriented website with information about sex, health, and relationships). Much of this information, however, is provided in Swedish. Youmo.se is a site connected to umo.se with material targeting young migrants in the languages of the main migrant groups, including Arabic. The availability and content of SRH information in different languages by local health authorities differ between the 21 regions in the country.

Sexual education is provided in primary and high schools. There is no standard programme for sexual education given to migrants who do not attend primary and high schools; however, there are ad-hoc projects in different regions. Interpreting services during healthcare visits are provided free of charge for migrants who cannot speak Swedish upon their request (Förvaltningslag § 13 (2017:900), [Administration Act], 2017; Smittskyddslag (2004:168), 7 kap. Ersättning, [Communicable Diseases Act], 2004).

SRH services related to contraception, abortion, pregnancy and delivery, as well as testing and treatment for sexually transmitted infections that are of ‘public health importance’ are free of charge and can be accessed without a social security number by, for example, undocumented migrants and asylum seekers. In addition, youth clinics, which are a comprehensive network of preventive SRH services oriented to youths (upper age limits around 24 years old, although they vary from clinic to clinic), can be accessed by all young people with or without a social security number (FSUM [Föreningen för Sveriges ungdomsmottagningar], 2018). Other services such as consultations related to gender identity, sexual function, and infertility are subject to the same subsidised service fees for people with a social security number, while people without a number are not entitled to such services (Socialdepartementet, 2010). In addition, several private digital clinics provide services related to SRH, including consultations on sexual function. These services are also publicly funded and accessible to people with a social security number only.

### Study design and participants

For this qualitative interview study, we recruited the participants using two different methods; snowball sampling using personal contacts; and through an announcement on Facebook. This allowed us to reach more potential participants and widened the type of experiences discussed in the interviews. The potential participants were approached first with oral and written information about the study, including a consent form. If the participant agreed to the interview, a time for the interview was booked for another occasion. We recruited 13 participants who identified themselves as men, aged 22 to 37 years, speak and understand Arabic, and had moved to Sweden within the last 10 years. All respondents held a residence permit and were living in different geographical areas in Sweden. Respondents had various countries of origin (six from Syria, three from Palestine, two from Iraq, one from Yemen, and one from Iran). Two of the participants referred to themselves as Kurdish and the others as Arabs. Eight participants had a higher education degree, while five did not. Nine participants had visited SRH services in Sweden at least once, while four have never visited SRH services in Sweden. All the participants identified themselves as cis and heterosexual.

### Data collection

All the interviews were conducted in Arabic by the first author, whose mother tongue is Arabic. We asked the participants to complete the interviews through telephone or video calls, since at the time when the interviews were conducted, travel was not recommended due to the COVID-19 pandemic. All participants preferred audio rather than video. The interviews were carried out between December 2020 and March 2021. The interviews were semi-structured and lasted between 32 and 57 min (43 min on average). We first piloted the interview guide and adapted its questions and the probing questions. The interview guide consisted of broad questions about the meaning of SRH for the participants; the needs that migrant men have regarding sexual and reproductive health and rights (SRHR); the experiences, perceptions, and challenges that the participant and other migrant men have when seeking SRH services; and how to improve theses services for migrant men (full interview guide is attached in Appendix 1). The participants’ answers, and opinions were elaborated with probing and follow-up questions. The interviewer repeatedly summarised the answers and asked the interviewee whether he thought the summary adequately conveyed his answers, which allowed the participant to provide clarifications and further elaborations of his opinions when needed.

We followed a pragmatic approach, as recommended by Braun and Clarke in relation to reflexive thematic analysis (Braun and Clarke, 2021b), to determine the number of interviews taking into consideration the norms of qualitative health research, the process of analysis and the time and resource constrains. Although this paper is built only on thirteen interviews, each of these interviews provides rich and deep insights on the topic. Besides, the fact that we collected and analysed data concurrently and that we used theoretical concepts in the analysis allowed us to develop and refine the themes progressively. We stopped this process when we were satisfied with the structure of themes developed which allowed us to answer our research questions.

### Data analysis

The interviews were recorded and transcribed verbatim with the help of a professional transcribing company and then checked by the first author. The analyses and data collection were performed in parallel following an emerging design and revising the research questions and preliminary analysis. Data were analysed using reflexive thematic analysis as described by Braun and Clarke (Braun and Clarke, 2006, 2021a). We used Microsoft word and excel to help us manage the process of handling transcripts, coding and the organization of the codes in candidate themes. In the first step, the first author reviewed the transcriptions by listening to the audio files of the interviews to check the quality of the interviews and familiarise himself with the material. He also translated parts of the interviews into English to facilitate the understanding and discussion of the research material with the other members of the research team who did not understand Arabic. The research team then read the translated materials and discussed the preliminary ideas. In the second step, the first author coded the interviews. Coding was close to the text and done in English to allow for a continuous discussion within the team of the coding process and the development of themes. In the third step, the research team discussed the codes and developed candidate themes. In the following step, the first author started to write the description and the arguments of the candidate themes. This was continuously discussed within the research team, which allowed for negotiating, refining, and coming up with the final four themes. This step followed an abductive approach allowing to go back and forth between the initial codes and the developed themes. In the final step, we selected the quotes and then built up and presented the final themes in this manuscript. Although most of the codes were on a semantic level and the analysis was done inductively with an experiential approach, we tried, when applicable, to identify latent meanings and underlying ideas in the empirical data.

### Ethical consideration

All potential participants were called prior to the interview and were given written and oral information about the study, including why the interviews were to be recorded and how the data will be handled. We emphasised the confidential and voluntary nature of the study. Oral consent was obtained on the day of the interview and recorded in a separate audio file. Ethical approval was granted by the Regional Ethics Committee in Umeå Dnr 2020–02,816.

## Results

**Fig. 1:**
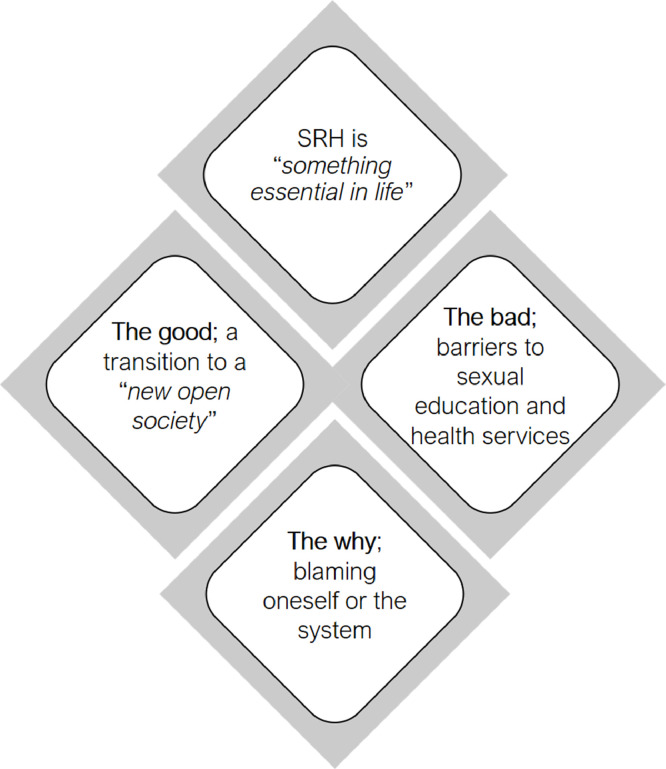
Arabic-speaking migrant men's perceptions and experiences of SRH services in Sweden.

In the final step of the analysis, we developed four themes (Fig. 1): (1) SRH is ‘something essential in life’; (2) the good: a transition to a ‘new open society’; (3) the bad: barriers to sexual education and health services; and (4) the why: blaming oneself or the system. The first theme describes how the participants perceived SRH as essential in their lives and as a means to build a family, as well as how they expressed the importance of being able to fulfil their rights to sexual education and SRH services. In the second theme, the participants explained the positive effects of moving to Sweden, which was perceived as an open society where SRH is dealt with as something ‘normal’. They considered that this new environment influenced migrant men's health-seeking behaviours positively. Despite these positive views, the participants pointed out several barriers migrant men face in accessing SRH services in Sweden, such as lacking sexual education, language difficulties, long waiting time, lack of holistic care, and not prioritising men's SRH. These barriers led to unmet SRH needs and are described in the third theme. Finally, the fourth theme centres on participants’ reasoning of the mismatch between being in this ‘new open society’ represented as full of possibilities (‘the good’), while at the same time, migrant men did not always have their SRH needs fulfilled (‘the bad’). Some participants justified this mismatch by not feeling entitled to ask for help and blamed themselves for not being prepared or serious enough when asking for help. In other instances, the mismatch reflected the flaws of a system that was portrayed as not responsive to their needs and reproducing cultural racism.

### SRH is ‘something essential in life’

This theme examines the value of SRH in the participants’ lives and how they define SRH. Most of the participants stressed the importance of SRH and described it as ‘something essential in life’ (participant E) and a basic life need. SRH and sexual relations are perceived to be a means for building a family, which is considered crucial and a norm. Some participants argued that marriage and building a traditional family were more important in the Middle Eastern societies from which they came than in Sweden. The ability to have children is therefore important and something that gives stability to marriage and life. Manhood is perceived to be threatened when the ability to have sex and reproduce is affected. In fact, some participants equated manhood with being fertile and centred SRH around the ability to beget children.

‘In our society, you are healthy if you don't have infertility. Other than infertility, nothing is considered SRH…Sexual pleasure and even childbirth are not considered as sexual health.’ (participant C)

SRH was also considered to go beyond physical health to include mental health, which was believed to strongly affect sexual health and sexual desire. The participants perceived migrants as especially exposed to mental health problems and in need of mental health support. For example, stress and depression, which could affect sexual life and sexual performance, might be related to the migration process, the lack of social relations, and other life situations, such as unemployment, as participant M argued:

‘Of course, relieving the mental stress of migrant men is the most important thing, when there is mental stress due to unemployment of migrant men… I am not working. I can't afford to have a child. I should be self-reliant. I mean, I should work and be able to meet the needs of my house and my child before thinking about having the child. I do not want to rely on the social services.’

The participants contrasted how in the societies from which they come, sexual satisfaction and pleasure were not considered as important as building a family (see, for example, the previous quotation from participant C). However, there were also references to the importance of sexual satisfaction and sexual pleasure in the interviews:

‘It is very important for men to understand their and their partners’ needs in order to satisfy them and to have harmony in life in general.’ (Participant F)

Building upon this perceived importance of SRH, the participants highlighted the need of newly arrived migrants to receive sexual education and get access to SRH services that allow them to ensure good and satisfactory SRH. More specifically, they argued that new migrants need to get general information about SRHR, family planning, how to protect themselves from STIs, where to get help for SRH problems, and rights and obligations. For them, sexual education and SRH services are rights that every person should enjoy:

‘Healthcare is a right for everyone in the country… and you should have the right to reach it in a comfortable way without difficulties searching for it. It should be available without obstacles — language, finances, residence permit, sex, nothing. It should be available in all ways.’ (participant E)

So, SRH is important, and participants articulated that this importance justifies the existence of a network of sexual education and services, and that such a network should reach everyone, independently of gender, language, and socioeconomic or legal status. The next themes dig deeper into how participants perceive that the ‘new’ Swedish context provides such a network of services but fails to reach everyone, and the perceived reasons for such a contradiction.

### The good: a transition to a ‘new open society’

In this theme, the participants explain the positive effects of moving to Sweden. Comparing to their countries of origins, they perceive Sweden as an open society with greater variety of services related to SRH, and where people talk about sex freely and SRH is considered as any other health issue.

Generally, the participants felt respected, listened to, and cared for in the Swedish SRH services. Comparing to their countries of origin, some participants considered the Swedish healthcare system better and easier to get help from. Additionally, the Swedish healthcare system offers a greater variety of services related to SRH that are not available in their countries of origin, such as antenatal classes, postnatal visits, and digital clinics, which allow for more confidentiality and privacy. This facilitates the access to SRH services in Sweden. However, more than resources, the participants explained how coming into what they considered a ‘new open society’, in relation to SRH, positively affected their perceptions, attitudes, and behaviours in relation to SRH services.

The perception of Sweden as an ‘open society’ has to be understood in contrast to how participants described the cultural and gender norms in their countries of origin as hindering help seeking for SRH-related issues. The cultures where migrants came from are described as closed and silent cultures in relation to sex and sexual problems; sexual topics are accompanied with shyness, shame, and taboo. For men, sexual problems are perceived as a threat to masculinity, which makes them hesitant to talk about their sexual problems and try to find solutions themselves rather than seeking healthcare. In contrast to their countries of origin, the participants described Sweden as a ‘new open society’ where sexual information is available and people know more about SRH and are more open to discuss sex and sexual life freely as a normal part of life.

‘Here there is more openness…more freedom in stuff related to sex...everything is permissible. You can talk and ask a doctor about it…there [Arab countries], there is shyness. Even if you have a disease, you don't visit a doctor… Here [Sweden] you can go and get treatment with privacy… you don't feel shy...You feel it as any other medical condition.’ (participant G)

As participant G described, being in this new context leads to changes in health-seeking behaviour. The open environment and the privacy that participants described as characteristics of the Swedish healthcare services make it easier for migrant men to discuss their sexual life more comfortably and be more open about their problems and more prone to seek healthcare. However, the participants considered that such changes in behaviours/attitudes did not happen equally to all migrants. For example, participant A explained:

‘Those who live alone in groups and they don't mix with others, those, surely, did not change. Those live geographically in Sweden but culturally, they are still living in their neighbourhood…of their home country, I mean.’

In this quote, the participant considered that the more open to the new society the migrants are, the more likely they are to change their attitude and behaviours. Several participants also mentioned that the openness to the new society and thus the changes in health-seeking behaviours were facilitated by being younger, having more contact with Swedish society, and longer periods spent in Sweden.

### The bad: barriers to sexual education and health services

Even though the participants expressed a general contentment with the Swedish healthcare system in relation to SRH compared to that of their countries of origins, they also discussed how they faced several barriers to access sexual information and other SRH services. Most of the participants indicated that they use the Internet to access sexual information, and they expressed a challenge in finding reliable sexual information in Arabic. While some used 1177 (the official Swedish healthcare guide website) and other reliable public websites, they indicated the need for a high level of command of the Swedish language to understand the medical language used. Many, therefore, used Google to search for information in Arabic and remained sceptical of the reliability of the information they obtained; as participant A articulated: ‘Google is not reliable, and anyone can write whatever he wants and publish it’.

Besides affecting the ability to get reliable sexual information, the participants indicated that the language barrier could affect communication with HCPs. The use of interpreters as a solution to the ‘communication problem’ was perceived as problematic since (1) it might be embarrassing to communicate sexual health issues through an interpreter, and (2) they felt uncertain about the reliability of interpreting. As participant I further elaborated:

‘The person is saying something and the interpreter is saying something different… these things are very important…the language is very important for the user so he can by himself communicate his symptom, his feelings…Most of the interpreters do not interpret well… Why? Maybe because of the dialect?’

Not speaking Swedish was thus perceived as a barrier for accessing information and healthcare. In addition, participants complained that new migrants were provided with formal sexual education only if they were young enough to study in the Swedish high schools. This was perceived as a problem since migrants in general may have not received sexual education in their countries of origin and therefore might be less informed of SRH, as participant K articulated:

‘Here they learn about these things [sexuality and SRH] in high schools, but those who are older don't study this. So I missed it because I am one year older…so I was forced to study these things alone since there is no other way to learn.’

When it came to accessing SRH care services, participants also pointed out several barriers. Most of the participants considered the long waiting time in general, especially while waiting to see a specialist, as a main barrier to receiving healthcare in Sweden. During this waiting time, participants do not usually receive any treatment or advice.

‘Unfortunately, it was almost six months or more until I got an appointment… I mean six months…people could commit suicide…I had erectile dysfunction but did not totally lose erections, but other men might have no sex at all… They could commit suicide while waiting for six months… This is wrong. I think those who have sexual problems should have priority… They can't wait six months or more. They should be treated as soon as possible. (participant G)

Besides the long waiting time, most of the participants who sought help for sexual health issues complained that the visits went without any physical examination or investigations and that the HCPs did not try to arrive at a diagnosis; rather, they rushed to treat the symptoms and did not follow up the problem. All of this led the participants to perceive that the healthcare system in Sweden was not taking the participants’ sexual health problems seriously. This perception was reinforced by the lack of seeing the patient as a whole and neglecting mental health aspects:

‘He [the doctor] did not discuss things with me and reach a diagnosis… [he did not say] you're mentally tired or something, no…he directly gave me the treatment. (…) they don't deal seriously with the disease.’ (participant G)

Another complaint about the Swedish healthcare system was that some participants considered themselves excluded as partners during antenatal care or other SRH-related visits. Men acknowledged that pregnancy and giving birth are about the women's body, and therefore it is normal to focus on the women, but they also complained that they are not included in the conversation with the HCPs. While both partners received information about the pregnancy, birth, and child's health, men stated that there was no information for them about contraceptives, family planning, or how to support their partners during pregnancy.

‘I think men should have an equal role to women in things related to pregnancy and child care... the same importance, although he is not the one who is living the experience, but at least he should have more knowledge about the topic.’ (participant L)

Participants also criticised the way the healthcare system is organised, and the interventions that were prioritised did not always align with the SRH needs they felt. One example put forward was sperm analysis. The criteria of getting a sperm analysis were perceived to be very difficult and ‘non-logical’. Participant A, who had a history of being divorced due to infertility and was denied sperm analysis, explained his need: ‘I have a future plan to have a relationship, but I am hesitant because I do not want to enter a relationship and fail, and it affects me negatively’.

All of the previous factors led some participants to lose hope in the system and to seek help outside the healthcare system in the black market or outside Sweden. The black market was perceived as a common place to seek help for sexual dysfunction, as one participant who works in healthcare indicated. According to him, some migrant men consider the black market to be more convenient and less expensive, since Viagra is not subsidised. Participant H described his loss of hope in the Swedish system and his attempts to seek help outside Sweden:

‘I feel despair towards Sweden and the healthcare system in Sweden… they underestimate the real disease…you found them not interested in the person… they only care about the time they spend on you listening about your problem. (…) I am contacting doctors outside Sweden, but they want me to go there so they can help me… but here in Sweden I actually don't know what to do.’

### The why: blaming oneself or the system

In this theme, we present how the participants explained the mismatch between the ‘new open society’, which is supposed to make it easier to seek and get information, education, and healthcare, and the inaccessibility and despair some of them experienced in relation to education and services related to SRH. On the one hand, some participants justified this mismatch in terms of them not being entitled to receive help or to criticise the system. For them, they live in a foreign country that welcomed them, so they should take only what they are offered and not ask for more. Participant C explains how he had no expectation: ‘I was not waiting for anything because I am living in a country that is not mine’. Therefore, he was content with the healthcare he received. Participant D further explains how migrant men should not criticise the system:

‘I don't think migrant men need anything. (…) A country that opened its doors to us. We should respect everything in it…I mean in your country, you were not asking why did you start asking here that you want this and this.’

Besides not being entitled to ask for help, some participants felt that they did not receive the help they expected simply because of their bad luck, as participant B articulated:

‘I remember that time I went to the health centre… Honestly, I can say to you it was as if I did not go anywhere… I mean I did not have a diagnosis, treatment…nothing. (…) Honestly, I don't know why…maybe I went at the wrong time or my bad luck.’

Some participants also reasoned that they did not receive help because they had not taken enough responsibility for their diagnosis, and they blamed themselves for not presenting their problems with the severity they required. The participants felt they need to read more about their cases and tell the doctor all their symptoms/story even without being asked. Some felt they need to be angry and insist on performing a physical examination and investigations; otherwise, they will not get a diagnosis or treatment, and the doctor will not respond to their concerns.

‘I went back. I talked with a third doctor. Of course, this time my attitude was a little angry, let us say, from my experience with the two former doctors. So he cooperated with me more because I was very serious about the issue. I will not allow anyone to say that I have no problem from only looking at or talking to me. (…) I collected all the information that I wanted through my personal efforts. I read many articles, so, let us say, that I told the doctor what my problem was. (participant A)

The participants discussed general problems in the health system such as long waiting times, women-centred care, and a lack of holistic care, as mentioned in the previous theme. However, the participants also reflected about being Middle Eastern men facing the Swedish system and blamed the system for cultural racism. From this standpoint, participants did not blame themselves or bad luck for not getting the services they needed and wanted, but instead used arguments that highlighted that the system was specifically failing them. These arguments included criticisms of the system for not providing sexual education. Moreover, education in general was perceived sometimes as a way of indoctrinating migrants on how to be good ‘Swedish’ citizens and portraying them as lacking and deficient, which makes them feel othered and resistant to accept new information. The participants emphasised that in order for sexual education to be acceptable, it should be delivered in a culturally sensitive way, preferably through cultural mediators who understand and respect migrants’ cultures.

‘Honestly, we all have noticed how they don't consider our backgrounds during the Swedish society orientation course. The information they give does not reach us in a way that we can accept and adopt… here they teach us in a way that ‘you should be like this’, ‘you should do this’…Generally, no person will accept a change when you force it on them.’ (participant K)

Some participants argued that the adverse treatment they have received was due to the lack of experience or professionalism of the HCPs. However, for other participants, these behaviours were related to racism. For example, participant I argued: ‘Maybe because we look different we receive different treatment’. Participants described how HCPs sometimes used cultural stereotypes that portrayed migrants as faking symptoms or exaggerating to deny them services:

‘I went to the first doctor and was telling him that I feel I have a problem with the testosterone, and I want to test myself. The doctor answered by saying, ‘You all say that. You have nothing’, and the visit finished. Who are those in ‘you all’? I don't know (laughter). He said, ‘You all say that’. (…) I don't like to use the word racism, but, yes, there is a possibility that the doctor might not take you seriously… might not listen to you.’ (participant A)

In the previous theme, we described how some participants felt excluded from the conversation when they visited the HCPs together with their partners. Some participants argued that such exclusion was based on the stereotypes that some HCPs have about Middle Eastern men as controlling and anti-gender equity:

‘The talk was not oriented to me…I was an accompaniment... there was no discussion or information oriented to me…this is one of the negative things… there should be information for both. (…) Maybe they are focusing on the migrant woman more than the man due to women's rights being high priority here…and maybe they are saying: the Eastern man is controlling, so he does not need [help].’ (participant I)

## Discussion

Our results describe how SRH played an essential role in the participants’ life and how sexual information/education and SRH services were perceived as needs and rights. The participants, therefore, were content with the new possibilities and the ‘new open society’ that living in Sweden provided. However, these possibilities were not equally offered to all. Sexual education was not available for all migrants, only those attending primary and high schools, and education provided to migrants in general was perceived as indoctrinating. Several barriers contributed to deprive some participants of their rights to SRH services, including long waiting times, a lack of prioritising their needs, women-centred care, and a lack of holistic care. The participants explained their adverse experiences in various ways. They sometimes argued that these experiences might be random or that they failed to express their needs (i.e., they blamed themselves). Other times they argued that their experiences indicate cultural racism within the SRH services.

Although the participants conceptualised SRH putting a focus on the ability to have children and build a family, they also highlighted several aspects of SRH that expanded beyond physical health and reproduction and are in line with comprehensive definitions of SRH (World Health Organization, 2017). The participants also highlighted the importance of fulfilling their rights to sexual education and SRH services on equal terms, which is highlighted as a priority in several national and international strategies (Folkhälsomyndigheten, 2020; Starrs et al., 2018). The recognised importance and value of SRH in the participants’ lives challenge the stereotypical portrayal of migrant men as not knowledgeable and not interested in SRHR that can be found in certain media and political discourses in Sweden. Other researchers have problematised the dominance and effects of such stereotypical discourses on migrant men (Edenborg, 2020; Herz, 2019).

With the importance given to SRHR, the participants appreciated the ‘open’ society and the opportunities that the organisation of the welfare system in Sweden offer for fulfilling their SRHR. This is not surprising, since, on a global scale, Sweden is a forerunner in legal frameworks that promote gender equity and LGBTQ rights. On the other hand, it is important to acknowledge that, even in Sweden, there is room for improvement. For example, intimate partner violence in Sweden remains high compared to other European countries (Gracia and Merlo, 2016), and the MeToo movement showed the pervasiveness of sexual violence and harassment in Sweden. Besides, studies show that alongside the ‘openness’, ‘traditional’ sexual scripts and strong heteronormative values coexist in Swedish society (Linander et al., 2021; Odenbring, 2019).

Despite the appreciated opportunities in the welfare system of Sweden that helps the participants to fulfil their SRHR, they pointed out that they face challenges in their access to sexual education in Sweden. Comprehensive sexual education is only offered in primary and high schools, and there are ad-hoc attempts to provide some health information during the civic orientation course, which sometimes covers areas of sexual health (Al-Adhami et al., 2021; Svensson et al., 2017). Other studies have reported that these attempts were valued by migrants as empowering and helped them to get useful information, adopt healthier habits, and gain insights on ‘the Swedish values and society’ (Al-Adhami et al., 2021; Svensson et al., 2017). Our study, although not evaluating a specific programme or ad-hoc education, highlights the need for sexual education for migrants and the concerns about the way such education will be delivered. These concerns were also raised by Arabic-speaking parents about the sexual education of their children in the Swedish schools, where the parents discussed the clash between their values and Swedish values (van Wees et al., 2021).

Furthermore, the participants pointed out several adverse experiences in the SRH services that indicate a lower priority for men's SRH services, including feeling excluded in antenatal care, a lack of holistic care, and long waiting times for specialised care. Similar adverse experiences are reported in the literature among men in Sweden (Baroudi et al., 2021). The exclusion of men in healthcare related to reproduction is a common experience among men in Sweden (Christianson et al., 2013; Widarsson et al., 2012). The lack of prioritising men's SRH is exemplified by the lack of medical training about men's sexual health among nurses and midwives and the low organisational support to provide SRH services for men (Grandahl et al., 2019; Klaeson et al., 2017). The lack of holistic care and overlooking the emotional and psychological aspects of care are also highlighted in SRH services in Sweden, such as infertility and antenatal care (Sylvest et al., 2016; Widarsson et al., 2012).

Moreover, the participants highlighted inequities that they interpreted as based on their migration background, ethnicity, and racialisation. The participants highlighted experiences of being treated differently due to how they looked, being negatively stereotyped as controlling Middle Eastern men, and being accused of faking or exaggerating symptoms. All these experiences could be explained by cultural racism (Taguieff, 2001). Through exclusion, migrants’ appearances, behaviours, and languages/accents are differentiated as something deviant and ‘inferior’, creating a way of othering migrants and justifying excluding them from getting the help they need (Daun, 2008). At the same time, it is difficult to point out racism as a reason for such experiences, since racism, based on racial differences, is generally thought by Swedish society to be overcome in the ‘exceptional’ Swedish culture. However, these inequities are still reproduced and sustained in the form of cultural racism, based on cultural rather than racial differences (Ahlberg et al., 2019). In addition, this internalised feeling of underserving-ness/unworthiness of help can be interpreted as internalised racism, where the participants consciously or unconsciously fail to problematise discrimination and thus contribute to endorse the racial/cultural hierarchy (Pyke, 2010). Although the participants are reproducing the dominant culture's power, internalised racism should be understood as a product of cultural racism practiced over them (Pyke, 2010).

Men's SRH problems are usually discussed as a product of masculinity norms which might increase their risk-taking and affect their health seeking behaviours (Shand and Marcell, 2021). In contrast, the organisation of the health system such as lack of available “male-friendly” facilities, low engagement of men in SRH, low prioritization of men’ SRH in healthcare and in research, and cultural racism in healthcare and society are rarely highlighted (Ahlberg et al., 2019; Baroudi et al., 2021; Nyalela and Dlungwane, 2022). It is necessary to explore other structural factors that men in general and Middle Eastern migrant men in particular face when seeking and receiving SRH services. Therefore, there is a need to provide comprehensive sexual education for all, strengthen SRH services provided to men, and develop an action plan to reinforce the anti-discrimination/racism policies in healthcare and society.

### Methodological consideration

Individual interviews were considered more appropriate to enable the participants to speak comfortably and more freely, since the topic might be considered by the participants as more sensitive to be discussed in groups. We acknowledge that those who participated may have had more interest and experience in the topic than other Arabic-speaking migrant men. Although this can be perceived as a potential limitation, this might have also contributed to the richness and depth of our data. Also, this study explored cis-men's and heterosexual men's experiences, which might differ from the experiences of other groups of migrant men, such as transmen, men who have sex with men, and gay men. Even though we did not exclude these groups in the recruitment phase, all the participants identified themselves as *cis* and hetero.

Furthermore, we choose to deal with Arabic-speaking men as a group coming from similar cultures. While there are similarities between Arabic cultures, dealing with them as essentially similar might mask the differences between these cultures and thus the differences in experiences this group of migrants faces. Furthermore, the participants are a heterogeneous group with different ages, stages in life, life circumstances, levels of education, and lengths of stay in Sweden; therefore, they might have various understandings of SRH and different experiences of SRH services. Despite this heterogeneity, young migrants coming from the Middle East are often portrayed by part of the Swedish society and media as a ‘homogeneous’ group characterised as sexual perpetrators and a threat to Swedish society, culture, and values (Herz, 2019; Horning et al., 2020). We acknowledge that focusing on them as a group under the ‘common’ ground of sharing a language risks reinforcing the idea of a ‘shared identity’ that downplays the heterogeneity of this group and risks stereotypification.

The first author is an Arabic-speaking man born in Syria. This facilitated access to and trust from the participants. However, the participants spoke various dialects of Arabic, which may have influenced the familiarity with the language during the interviews, and we acknowledge that we may have failed to capture possible misunderstandings. However, the first author tried to frequently summarise and rephrase the participants’ responses and asked for confirmation and explanation.

## Conclusion

This article explored Arabic-speaking migrant men's perceptions and experiences of receiving sexual education/information and SRH services. Sexual education was mostly unavailable to migrants, and existing education in general was perceived as indoctrinating migrants on how to be good citizens which created a barrier for migrant men to fulfil their needs. Although the participants described being generally content with the new environment and the available SRH services, they experienced adverse treatment that can have two explanations: a lack of prioritising men's SRH in the Swedish health system and cultural racism. These challenges created a great barrier for migrant men to receive adequate/acceptable SRH services. Comprehensive sexual education should be provided for all and an action plan to help apply the anti-discrimination policies in healthcare should be developed.

## Declaration of Competing Interest

None to declare.

## Appendix Supplementary materials

Image, application 1
