# Supplementary material for: The good, the bad, and the why: How do Arabic-speaking migrant men perceive and experience information and services related to sexual and reproductive health in Sweden?
Source: J Migr Health. 2023 Jan 29;7:100153. doi: 10.1016/j.jmh.2023.100153 (PMC9926105; doi:10.1016/j.jmh.2023.100153)
Supplement: Supplementary file 1 [file mmc1.docx]

# Interview Guide

**Introduction**

The interviewer introduces himself and the project, and makes sure that the participant has understood the purpose of the study and consented to participate.

**Background information**

1. Short presentation of the informant’s gender, age, country of birth, background, how long he has been in Sweden and where he lives in Sweden.

**Thematic interview part**

Follow-up questions: can you tell us more? How do you mean? Can you clarify?

### Theme: sexual and reproductive health

2) What does sexual and reproductive health means to you?

3) How has your view about sexual and reproductive health changed since you came to Sweden?

4) What needs for information/knowledge/services do you think exist among migrants in the field of sexual and reproductive health?

5) What is the most important source of information for you in sexual and reproductive health?

### Theme: the meeting in the healthcare services

6) What experiences do you have of the sexual and reproductive health services in Sweden? (Primary care, youth clinic, school health care, maternity care, specialist care…)

7) Do you know where to find help regarding sexual and reproductive health?

8) How do you feel about seeking help for sexual and reproductive health?

(How do the legal, social and economic contexts in Sweden affect migrants' ability and willingness to seek help?

(How do gender norms and roles affect migrants' ability and willingness to seek help?)

9) What responsibility do men and women have when it comes to using contraceptives or seeking help for SRH issue?

10) What expectations do you have from the Swedish sexual and reproductive healthcare?

11) What challenges do you/other migrants face in sexual and reproductive healthcare?

12) How can we improve sexual and reproductive health among migrant men in Sweden?

**Closing**

13) Do you want to add or ask something? Have we forgotten something important?

14) How have you experienced this interview and talking to me about SRHR?
